# Effects of assisted reproductive technology on severe maternal morbidity risk in both singleton and multiple births in Korea: A nationwide population-based cohort study

**DOI:** 10.1371/journal.pone.0275857

**Published:** 2022-10-10

**Authors:** Jin Young Nam, Seoyeon Hwang, Sung-In Jang, Eun-Cheol Park

**Affiliations:** 1 Department of Healthcare Management, Eulji University, Seongnam, Republic of Korea; 2 Department of Preventive Medicine, Yonsei University College of Medicine, Seoul, Republic of Korea; 3 Institute of Health Services Research, Yonsei University, Seoul, Republic of Korea; University of Illinois, UNITED STATES

## Abstract

**Background:**

Whether infertility treatment predicts severe maternal morbidity in both singleton and twin pregnancies is controversial. We conducted this nationwide population-based cohort study to compare pregnancies conceived through assisted reproductive technology treatments, such as intrauterine insemination or in vitro fertilization, with unassisted pregnancies.

**Methods:**

This study included 269,930 women who experienced childbirth in 2018, using data of the National Health Insurance Service National Delivery Cohort in Korea. The primary outcome was assessed using a severe maternal morbidity algorithm developed by the Centers for Disease Control and Prevention in the United States. A modified Poisson regression was used to estimate the adjusted risk ratio of severe maternal morbidity.

**Results:**

Severe maternal morbidity occurred in 6,333 (2.3%) of 280,612 deliveries investigated. The risk of severe maternal morbidity was approximately 1.5-fold higher among women who received in vitro fertilization (risk ratio: 1.51, 95% confidence interval: 1.36–1.68) than among fertile women. However, no significant association between intrauterine insemination and maternal morbidity was identified. Via subgroup analysis, in vitro fertilization increased the risk of severe maternal morbidity by 1.6- and 1.3-fold in singleton and multiple births, respectively (singleton: risk ratio: 1.62, 95% confidence interval: 1.43–1.83; multiple birth: risk ratio: 1.31, 95% confidence interval: 1.07–1.60).

**Conclusions:**

This study suggested that in vitro fertilization was associated with the risk of severe maternal morbidity in both singleton and multiple births. Further research should identify patient- and treatment-specific factors that may mitigate or prevent adverse maternal health risks.

## Introduction

Severe maternal morbidity (SMM) is defined as an unintended outcome of the process of labor and delivery that results in significant short- or long-term consequences to a woman’s health [1]. It is associated with increased medical costs due to prolonged hospital stays and increased family burden [2, 3]. SMM is highly preventable and has received a high level of attention globally, particularly in research, with the aim of reducing its incidence [3, 4]. If conditions underlying SMM are not identified and treated, they can potentially lead to maternal mortality [1]. Therefore, to improve maternal healthcare, additional efforts to reduce the rates of SMM are required. One strategy for reducing the rates of SMM is the identification of its potential risk factors.

Known risk factors for SMM, including older age, multiple births, and cesarean section delivery, are associated with the use of fertility treatments, including assisted reproductive technology (ART) [5, 6]. Previous research revealed that women who undergo ART are at an increased risk of placenta previa, morbidly adherent placenta, pregnancy-induced hypertension, placental abruption, blood transfusion, intensive care unit admission, gestational diabetes, and comorbidities and maternal mortality, compared with those who do not undergo ART [7–9].

It is well known that pregnancies conceived through ART are associated with increased risk of adverse maternal outcomes, a finding that is mainly due to the increased multiple pregnancy rate observed with ART. [10] The number of embryos transferred is associated with ART success [11]; however, single-embryo transfer or minimalizing-embryo transfer are popular and effective methods for avoiding multiple pregnancies in developed countries [12, 13]. Although a number of studies have explored the associations between ART and adverse maternal health outcomes globally, no studies have assessed the use of single-embryo transfer or the effects of ART on SMM in Korea.

In recent decades, Korea has faced serious problems associated with low birth rates and an aging population. As a country with the lowest fertility rate globally, Korea’s total fertility rate was its lowest ever measured in 2020, at approximately 0.84 [14]. To solve complex problems associated with low fertility rates, the Korean government has supported infertile couples to promote childbirth [15], including financial support or health insurance coverage for ART treatment from the government [16]. Over the last two decades, twin birth rates have rapidly increased, from five pairs per 1,000 births in 2000, to 18 pairs per 1,000 births in 2019 [17], and twin births have mainly occurred among women aged 30 to 39 years [17]. Twin birth and advanced maternal age may affect SMM and maternal mortality risk [18]. To better prevent adverse maternal health outcomes, this study aimed to explore the relationship between ART and SMM risk in Korean women.

## Materials and methods

### Data source and study population

This population-based study used data included in the Korean National Health Insurance Service Database (NHISD). The NHISD includes a health care utilization database, a national health screening database, sociodemographic information, and mortality data for the entire South Korean population [19]. The NHISD uses de-identified join keys rather than personal identifiers to link databases to ensure patient anonymity [19]. Healthcare utilization data, which comprises the largest component of data within the NHISD, includes information regarding inpatient and outpatient medical treatments (diagnosis according to International Classification of Diseases, 10th Revision [ICD-10] guidelines, length of stay, treatment cost, and services received), prescriptions (drug code, days prescribed, and daily dosage) [19], and healthcare provider information (types of healthcare institutions, human resources, and equipment for healthcare institutions) in Korea [19]. The study design was reviewed and approved by the Institutional Review Board of Eulji University (No. EU21-005). All methods were performed in accordance with relevant guidelines and regulations. The requirement for informed consent was waived by the Institutional Review Board of Eulji University in Korea because all analyses used anonymized National Health Insurance Service (NHIS) data.

The data of an NHIS delivery cohort were extracted from the NHIS claims database. The data included information on all childbirths that took place within South Korean health care institutions. This study defined childbirth using the diagnosis and procedure codes for pregnancies at a maternal age of 15–50 years, in which mothers gave birth in the hospital. The study population included mothers with childbirths that occurred between January 1, 2018 and November 19, 2018, as the NHIS began covering ART on October 1, 2017, and data collected 280 days before childbirth through six weeks post-childbirth were considered. In this study, childbirth was defined using any record of inpatient hospitalization, including a pregnancy-related diagnosis or procedure code for vaginal or cesarean delivery. The total study population included 298,563. The following exclusion criteria were applied: women who were hospitalized for more than 42 days; patients with childbirths that occurred after November 19, 2018; and those who had no healthcare institution data. In total, 269,930 births were included in the study.

### Severe maternal morbidity

To determine SMM, we utilized SMM algorithms developed by the United States’ Centers for Disease Control and Prevention (CDC). Women who were assigned at least one of the 21 previously established ICD-10 diagnosis or procedure codes during hospitalization for childbirth were considered. The SMM algorithm considers serious complications of pregnancy or childbirth, such as eclampsia or acute renal failure, and procedures performed in the treatment of serious conditions, such as blood transfusion or hysterectomy, which comprises 16 diagnosis codes and five procedure codes [20].

### Assisted reproductive technology

In this study, ART was defined as infertility treatment (ICD-10: N97) using any ART procedure, including intrauterine insemination (IUI) and in vitro fertilization and embryo transfer (IVF-ET). This database did not specify whether oocytes or embryos were autologous or from donors, since this is considered sensitive information. However, procedure codes for IUI or IVF-EF were included; therefore, women who underwent IUI or IVF-ET procedures and had successful childbirths were defined as those who conceived after ART treatment. Moreover, we defined the non-ART group as that comprising women who gave birth without undergoing any ART procedure.

### Covariates

Based on previous studies, several confounders were considered. Socioeconomic factors considered included maternal age (< 35 years, 35–39 years, ≥ 40 years); household income level (quartiles); type of insurance (self-employed insured, employee insured, and medical aid); and residential area (city, rural). Clinical factors, including mode of delivery (spontaneous vaginal delivery, instrumental delivery, cesarean section delivery); prenatal care (adequate, intermediate, inadequate) using Kessner’s Adequacy of Prenatal Care Index [21]; parity (nulliparous, multiparous); twin birth status (singleton birth, twin birth); and maternal comorbidities (0, 1, > 1) [22] were considered. Other covariates included hospital type, which was stratified based on the number of beds present (< 30 beds, 30 ≤ beds < 100, 100 ≤ beds < 500, beds ≥ 500).

### Statistical analyses

Distributions of the general characteristics of the study population were determined. The association between ART and SMM during hospitalization for childbirth was determined using a Poisson regression model with a robust error variance that was adjusted for all covariates, which estimated adjusted risk ratio (RR) and 95% confidence intervals (CIs). This model was generated to assess the relationship between twin birth status and SMM, which was stratified based on ART status. All statistical analysis was conducted using SAS (version 9.4; SAS Institute Inc., Cary, NC, USA). Values of P < 0.05 were considered significant.

## Results

The general characteristics of the study population according to ART status are shown in Table 1. Of the 280,612 women included, 7,254 (2.6%) underwent IVF-ET, 1,722 (0.6%) underwent IUI, and 271,636 (96.8%) did not undergo ART. Of the 2.3% of women had SMM, 448 (6.2%) of those who underwent IVF-ET, 73 (4.2%) of those who underwent IUI, and 5,812 (2.1%) of those who did not undergo ART had SMM.

**Table 1:** General characteristics of the study population.

	Assisted Reproductive Technology							
	Non-ART		IUI		IVF-ET		Total	
	N	(%)	N	(%)	N	(%)	N	(%)
*Outcome*:								
SMM								
No	265,824	(97.9)	1,649	(95.8)	6,806	(93.8)	274,279	(97.7)
Yes	5,812	(2.1)	73	(4.2)	448	(6.2)	6,333	(2.3)
*Confounders*:								
Maternal age (year)								
< 35	177,756	(65.4)	919	(53.4)	2,657	(36.6)	181,332	(64.6)
35–39	93,431	(34.4)	803	(46.6)	4,577	(63.1)	98,811	(35.2)
40 +	449	(0.2)	-	(0.0)	20	(0.3)	469	(0.2)
Income level								
Q1	55,823	(20.6)	266	(15.5)	1,324	(18.3)	57,413	(20.5)
Q2	61,058	(22.5)	277	(16.1)	1,066	(14.7)	62,401	(22.2)
Q3	96,333	(35.5)	670	(38.9)	2,498	(34.4)	99,501	(35.5)
Q4	58,422	(21.5)	509	(29.6)	2,366	(32.6)	61,297	(21.8)
Type of insurance								
Self-employed	54,681	(20.1)	211	(12.3)	1,044	(14.4)	55,936	(19.9)
Employees	214,921	(79.1)	1,509	(87.6)	6,194	(85.4)	222,624	(79.3)
Medical aids	2,034	(0.8)	2	(0.1)	16	(0.2)	2,052	(0.7)
Residential area								
Seoul	50,293	(18.5)	408	(23.7)	1,734	(23.9)	52,435	(18.7)
Metropolitan	70,535	(26.0)	456	(26.5)	1,936	(26.7)	72,927	(26.0)
Small cities	136,430	(50.2)	808	(46.9)	3,297	(45.5)	140,535	(50.1)
Rural	14,378	(5.3)	50	(2.9)	287	(4.0)	14,715	(5.2)
Mode of delivery								
Vaginal	64,639	(23.8)	284	(16.5)	832	(11.5)	65,755	(23.4)
Instrumental	85,074	(31.3)	451	(26.2)	1,441	(19.9)	86,966	(31.0)
Cesarean section	121,923	(44.9)	987	(57.3)	4,981	(68.7)	127,891	(45.6)
Preterm birth								
No	262,624	(96.7)	1,571	(91.2)	6,557	(90.4)	270,752	(96.5)
Yes	9,012	(3.3)	151	(8.8)	697	(9.6)	9,860	(3.5)
Prenatal care								
Adequacy	264,571	(97.4)	1,721	(99.9)	7,254	(100.0)	273,546	(97.5)
Intermediate	6,320	(2.3)	1	(0.1)	-	(0.0)	6,321	(2.3)
Inadequacy	745	(0.3)	-	(0.0)	-	(0.0)	745	(0.3)
Parity								
Nulliparous	140,917	(51.9)	1,369	(79.5)	5,759	(79.4)	148,045	(52.8)
Multiparous	130,719	(48.1)	353	(20.5)	1,495	(20.6)	132,567	(47.2)
Twin birth status								
Singleton	268,062	(98.7)	1,425	(82.8)	5,784	(79.7)	275,271	(98.1)
Twin	3,574	(1.3)	297	(17.3)	1,470	(20.3)	5,341	(1.9)
Comorbidities during pregnancy								
0	101,310	(37.3)	500	(29.0)	2,152	(29.7)	103,962	(37.1)
1+	170,326	(62.7)	1,222	(71.0)	5,102	(70.3)	176,650	(63.0)
Type of hospital (No. of beds)								
General hospital (500+)	16,077	(5.9)	297	(17.3)	1,441	(19.9)	17,815	(6.4)
Hospital (100–499)	19,952	(7.4)	218	(12.7)	1,140	(15.7)	21,310	(7.6)
Hospital (30–99)	139,580	(51.4)	839	(48.7)	3,284	(45.3)	143,703	(51.2)
Clinic (29 and less)	96,027	(35.4)	368	(21.4)	1,389	(19.2)	97,784	(34.9)

Abbreviations: ART, assisted reproductive technology; IUI, intrauterine insemination; IVF-ET, in vitro fertilization and embryo transfer; SMM, severe maternal morbidity

The association between ART and SMM, after adjusting for all covariates, is presented in Table 2. Women who underwent IVF-ET had an approximately 1.5-fold higher risk of SMM (RR: 1.51, 95% CI: 1.36–1.68) than those who did not. However, no statistically significant association between IUI and SMM was identified in this study. Compared with the non-ART reference group, SMM risk was elevated in those who were 35 years or older; received medical aids from government; underwent instrumental or cesarean section delivery; received inadequate prenatal care; were nulliparous; had twin births; and had maternal comorbidities. We also examined the association between ART and sub-indicators of SMM (S1 Table). Women who underwent IVF-ET had approximately 1.6-fold and 1.9-fold higher risks of blood product transfusion and sepsis, respectively (blood product transfusion: RR 1.59, 95% CI 1.42–1.78; sepsis: RR 1.89, 95% CI 1.29–2.77).

**Table 2:** Association between assisted reproductive technology and severe maternal morbidity, using unadjusted and adjusted models for all covariates.

	Severe maternal morbidity					
	Unadjusted RR	95% CI		Adjusted RR^a^	95% CI	
ART						
Non-ART	1.00			1.00		
IUI	1.98	(1.57-	2.50)	1.17	(0.93-	1.48)
IVF-ET	2.89	(2.62-	3.18)	1.51	(1.36-	1.68)
Maternal age (year)						
< 35	1.00			1.00		
35–39	1.32	(1.26-	1.39)	1.18	(1.12-	1.25)
40+	2.85	(1.95-	4.16)	1.83	(1.25-	2.68)
Income level						
Q1	1.05	(0.97-	1.13)	1.10	(1.02-	1.19)
Q2	0.97	(0.90-	1.04)	1.07	(0.99-	1.16)
Q3	0.99	(0.93-	1.06)	1.08	(1.01-	1.15)
Q4	1.00			1.00		
Type of insurance						
Self-employed	1.09	(1.03-	1.16)	1.10	(1.03-	1.17)
Employees	1.00			1.00		
Medical aids	2.04	(1.66-	2.50)	1.93	(1.56-	2.39)
Residential area						
Seoul	1.00			1.00		
Metropolitan	1.07	(0.99-	1.15)	1.18	(1.05-	1.34)
Small cities	1.08	(1.01-	1.16)	1.20	(1.09-	1.32)
Rural	1.09	(0.97-	1.23)	1.19	(1.03-	1.37)
Mode of delivery						
Vaginal	1.00			1.00		
Instrumental	1.67	(1.55-	1.81)	1.65	(1.52-	1.79)
Cesarean section	2.03	(1.89-	2.19)	1.62	(1.50-	1.74)
Preterm birth						
No	1.00			1.00		
Yes	3.62	(3.35-	3.91)	1.64	(1.51-	1.79)
Prenatal care						
Adequacy	1.00			1.00		
Intermediate	1.44	(1.25-	1.66)	1.33	(1.15-	1.53)
Inadequacy	1.93	(1.36-	2.73)	1.93	(1.36-	2.74)
Parity						
Nulliparous	1.29	(1.23-	1.36)	1.21	(1.15-	1.28)
Multiparous	1.00			1.00		
Twin birth status						
Singleton	1.00			1.00		
Twin	4.14	(3.77-	4.55)	1.48	(1.32-	1.65)
Comorbidities during pregnancy						
0	1.00			1.00		
1+	1.45	(1.37-	1.53)	1.29	(1.22-	1.37)
Type of hospital (No. of beds)						
General hospital (500+)	5.10	(4.77-	5.46)	4.18	(3.87-	4.51)
Hospital (100–499)	3.08	(2.86-	3.32)	2.85	(2.64-	3.09)
Hospital (30–99)	1.00			1.00		
Clinic (29 and less)	1.12	(1.05-	1.19)	1.17	(1.10-	1.25)

Abbreviations: ART, assisted reproductive technology; CI, confidence interval; IUI, intrauterine insemination; IVF-ET, in vitro fertilization and embryo transfer; RR, relative risk

a Adjusted for maternal age, income level, type of insurance, residential area, mode of delivery, preterm birth, prenatal care, parity, twin birth status, comorbidities, type of hospital, except for the analyzed itself.

**Fig 1:**
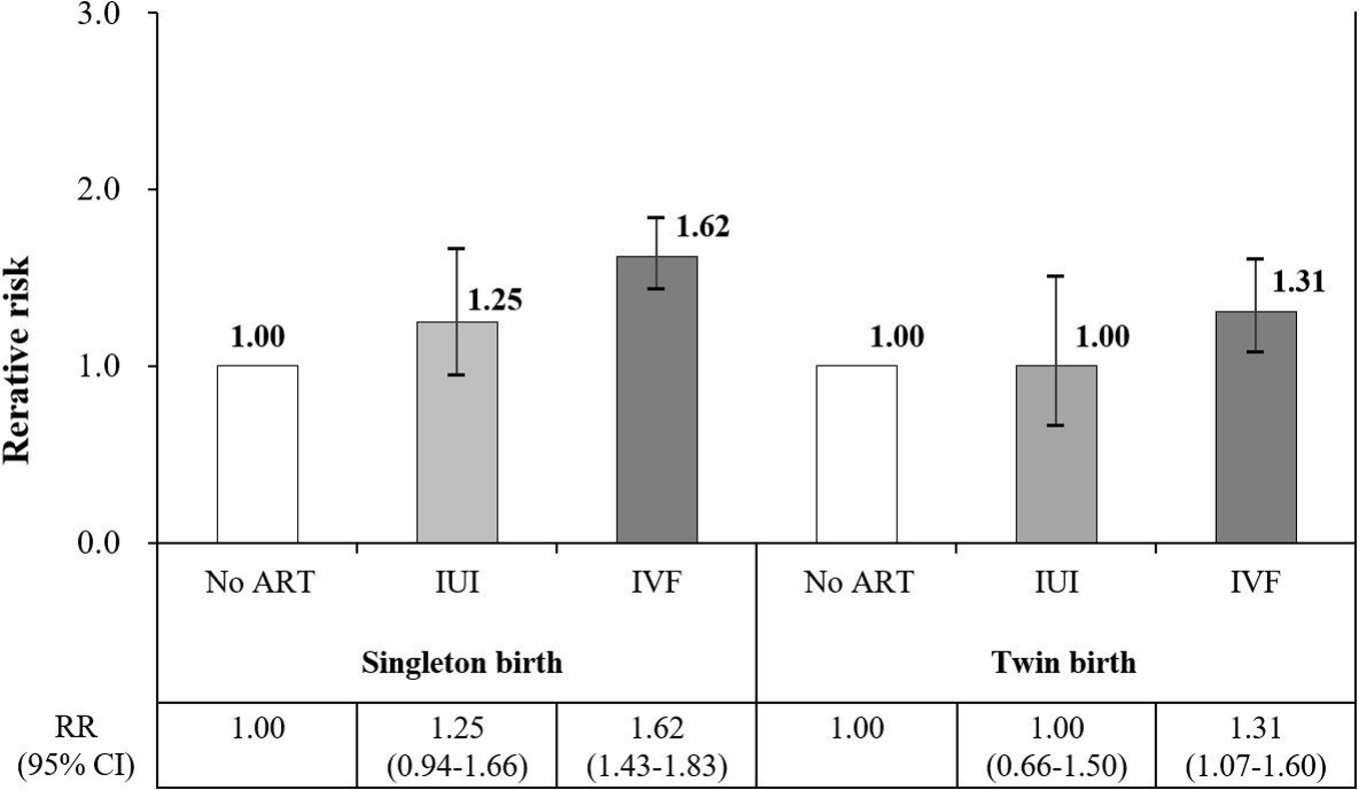
Analysis of the association between ART and SMM, stratified by twin birth status. Data are shown after adjustment for maternal age, income level, type of insurance, residential area, work status, mode of delivery, preterm birth status, prenatal care, parity, comorbidities during pregnancy, and hospital type. Abbreviations: ART, assisted reproductive technology; SMM, severe maternal morbidity; IUI, intrauterine insemination; IVF, in vitro fertilization; RR, risk ratio; CI, confidence interval.

An assessment of the effect of twin birth status on SMM, stratified based on ART status, was performed. In singleton pregnancies, SMM risk increased 1.6-fold in women who underwent IVF-ET, compared with those who did not (RR: 1.62, 95% CI: 1.43–1.83). No association between IUI and SMM was identified. Regarding twin births, the risk of SMM was 1.3-fold higher in women who underwent IVF-ET than in those in the non-ART group (RR: 1.31, 95% CI: 1.07–1.60); however, the association between IUI and the risk of SMM was not significant (Fig 1).

## Discussion

This study revealed that IVF-ET was associated with SMM; however, the association between IUI and SMM was not statistically significant. Moreover, IVF-ET increased the risk of SMM in women with singleton and twin pregnancies, while IUI was not associated with either of the groups.

Our finding that IVF-ET increases the risk of SMM, such as maternal complications and adverse outcomes, is consistent with those of previous studies [3, 6–9]. In particular, this work supports the findings of a Canadian cohort study, where women who underwent IVF were at an increased risk of SMM versus those who did not receive any treatment for infertility; however, there was no statistically significant association between IUI and any SMM, compared with the reference group [9].

We observed that the IVF-ET pregnancies were associated with higher SMM risk, compared with non-ART pregnancies in women with both singleton and twin births. It is well known that multiple births are associated with an increased risk of adverse maternal health outcomes. Further, our study findings were in agreement with those of a prior report showing an increased risk of preterm birth, cerebral palsy, and infant mortality in twin pregnancies [23]. The finding that twin births adversely affect maternal health outcomes among those with and without ART treatment remains controversial. One retrospective cohort study found no statistically significant association between ART treatment and SMM among women with multiple pregnancies, in part because multiple gestation itself increases the risk of many adverse conditions, including diabetes, hypertensive disorders, cesarean section delivery, abruption, and preterm birth [3, 23]. This may explain why women with both ART and non-ART multiple pregnancies are at an increased risk of SMM [3]. However, some researchers have suggested that previous studies may have been affected by statistically significant differences in obstetric risks, especially regarding rare outcomes [24]; statistical power affected the inclusion of an insufficient number of populations. Regardless of the mechanism by which multiple pregnancies are associated with SMM, findings suggest that minimizing multiple gestations is important to reduce the risk of SMM, which could be accomplished by elective single-embryo transfer with appropriate patient [3].

Furthermore, according to previous studies, singleton pregnancies after IVF are associated with increased adverse maternal outcome risks, compared with spontaneous conception [3, 8, 24], findings with which our study agrees. In a previous meta-analysis, women who conceived via ART treatment were shown to be at significantly increased risk of pregnancy-related hypertension, gestational diabetes mellitus, placenta previa, placental abruption, antepartum hemorrhage, postpartum hemorrhage, preterm birth, and other adverse maternal outcomes, compared with women who did not undergo ART with singleton pregnancies [24]. The mechanisms underlying the relationship between ART and poor maternal health outcomes in singleton pregnancies are unclear. One possible explanation of this finding is that ART procedures, maternal factors associated with infertility, or a combination of these increase the risk of adverse outcomes in ART pregnancies. Studies have shown that factors associated with ART procedures themselves including ovarian hyperstimulation and fresh versus frozen embryo transfer may increase the risk of adverse outcomes [9]. In addition, invasive infertility treatments may potentially cause maternal health problems that are increasingly harmful to women who undergo invasive procedures [9, 23]. In our study, women who received IUI, which is a noninvasive infertility treatment, were not at an increased risk of SMM, which might explain the association of invasive and noninvasive treatments with maternal health risk. Maternal factors associated with infertility were mentioned as a principal cause for increased SMM risk, rather than IVF treatment itself. One study observed that SMM and maternal mortality decreased from 2.17 to 1.39 after propensity score matching, suggesting that the association between SMM and infertility treatment is partially due to maternal age factors, generally maternal ages > 40 years, rather than the treatment itself [9]. Further research should be performed to clarify the mechanisms underlying the association between ART and obstetric risk.

This study has several strengths. First, it is the first study to assess the relationship between ART treatment and SMM risk using NHISD delivery cohort data. The NHIS delivery cohort includes all delivery cases in South Korea; therefore, the results of this study could represent the health status of all maternities in Korea. In addition, this study is the first study to identify a relationship between ART treatment and SMM risk after adjusting for multiple confounders including socioeconomic and obstetric factors via stratified analysis by plurality. We found that IVF treatment increased SMM risk in women with both singleton and multiple pregnancies.

This study has several limitations. Although this study included a large number of deliveries, the incidence of SMM and ART treatment was low. In particular, the subgroup analysis examining the effects of multiple births were limited by the low number of IUI and IVF-ET cases. In this study, we included women who delivered throughout a period of < 1 year, as ART treatment has been covered by NHIS only since October 2017; therefore, some analyses included a small number of cases, including those that involved the assessment of IUI or IVF-ET cases in women with multiple births. Despite the small number of individuals considered, we were able to confirm that IVF is associated with increased SMM risk. Further studies that include a larger number of cases are required to further investigate this association. Second, selection bias may have occurred, as only insured patients were considered. Therefore, uninsured women who received ART treatment were excluded. In South Korea, ART treatment is insured in women with appropriate maternal age and financial conditions to obtain ART treatment support. If women fail to meet specified conditions, ART treatment is not covered via the NHIS; therefore, they may have been included in the fertile women group in this study. This limitation has the potential to cause us to underestimate the association between ART and SMM risk; therefore, removal of this bias may show that ART is associated with an even greater risk of SMM.

In conclusion, this study revealed that women who received IVF-ET for infertility were at significantly increased risk of SMM versus those who were fertile; however, no statistically significant association between SMM and IUI was observed. In particular, women who received IVF-ET were at an increased risk of SMM when stratified by plurality versus fertile women. Further research that identifies patient- and treatment-specific factors that may mitigate or prevent adverse maternal health risks is required.

## Supporting information

S1 Table The association between ART and sub-indicators of SMM. (DOCX)
