# Supplementary material for: Effects of assisted reproductive technology on severe maternal morbidity risk in both singleton and multiple births in Korea: A nationwide population-based cohort study
Source: PLoS One. 2022 Oct 10;17(10):e0275857. doi: 10.1371/journal.pone.0275857 (PMC9550088; doi:10.1371/journal.pone.0275857)
Supplement: S1 Table — (DOCX) [file pone.0275857.s001.docx]

Table S1. The association between ART and sub-indicators of SMM.

| Sub-indicators of SMM |  | | Non-ART | IUI | | | IVF | | |
| --- | --- | --- | --- | --- | --- | --- | --- | --- | --- |
|  | N | (%) | RR | RR | 95% CI | | RR | 95% CI | |
| Blood product transfusion | 5084 | (1.8) | 1.00 | 1.27 | (0.99- | 1.64) | 1.59 | (1.42- | 1.78) |
| Sepsis | 715 | (0.3) | 1.00 | 0.45 | (0.11- | 1.79) | 1.89 | (1.29- | 2.77) |
| Disseminated intravascular coagulation | 569 | (0.2) | 1.00 | 0.41 | (0.10- | 1.67) | 1.33 | (0.89- | 1.99) |
| Pulmonary edema/ acute heart failure | 272 | (0.1) | 1.00 | 0.88 | (0.28- | 2.80) | 1.13 | (0.67- | 1.88) |
| Shock | 118 | (0.0) | 1.00 | - | - | - | 1.67 | (0.76- | 3.63) |
| Hysterectomy | 105 | (0.0) | 1.00 | - | - | - | 0.76 | (0.27- | 2.15) |
| Ventilation | 103 | (0.0) | 1.00 | 0.83 | (0.11- | 6.12) | 2.27 | (1.19- | 4.33) |
| Puerperal cerebrovascular disorders | 78 | (0.0) | 1.00 | 1.34 | (0.18- | 9.88) | 1.87 | (0.81- | 4.35) |
| Eclampsia | 60 | (0.0) | 1.00 | 1.51 | (0.20- | 11.39) | 0.67 | (0.15- | 2.91) |
| Conversion of cardiac rhythm | 46 | (0.0) | 1.00 | - | - | - | 0.41 | (0.09- | 1.83) |
| Adult respiratory distress syndrome | 35 | (0.0) | 1.00 | - | - | - | - | - | - |
| Acute myocardial infarction | 15 | (0.0) | 1.00 | - | - | - | - | - | - |
| Amniotic fluid embolism | 14 | (0.0) | 1.00 | - | - | - | - | - | - |
| Cardiac arrest/ ventricular fibrillation | 14 | (0.0) | 1.00 | - | - | - | - | - | - |
| Acute renal failure | 10 | (0.0) | 1.00 | - | - | - | - | - | - |
| Aneurysm | 4 | (0.0) | 1.00 | - | - | - | - | - | - |
| Severe anesthesia complications | 2 | (0.0) | 1.00 | - | - | - | - | - | - |
| Temporary tracheostomy | 1 | (0.0) | 1.00 | - | - | - | - | - | - |
| Air and thrombotic embolism | 0 | (0.0) | 1.00 | - | - | - | - | - | - |
| Heart failure/arrest during procedure or surgery | 0 | (0.0) | 1.00 | - | - | - | - | - | - |
| Sickle cell anemia with crisis | 0 | (0.0) | 1.00 | - | - | - | - | - | - |
